# Tuna Swarm Optimization: A Novel Swarm-Based Metaheuristic Algorithm for Global Optimization

**DOI:** 10.1155/2021/9210050

**Published:** 2021-10-20

**Authors:** Lei Xie, Tong Han, Huan Zhou, Zhuo-Ran Zhang, Bo Han, Andi Tang

**Affiliations:** ^1^Aeronautics Engineering College, Air Force Engineering University, Xi'an 710038, China; ^2^Unit 95806 of People's Liberation Army of China, Beijing, China; ^3^Unit 93525 of People's Liberation Army of China, Beijing, China

## Abstract

In this paper, a novel swarm-based metaheuristic algorithm is proposed, which is called tuna swarm optimization (TSO). The main inspiration for TSO is based on the cooperative foraging behavior of tuna swarm. The work mimics two foraging behaviors of tuna swarm, including spiral foraging and parabolic foraging, for developing an effective metaheuristic algorithm. The performance of TSO is evaluated by comparison with other metaheuristics on a set of benchmark functions and several real engineering problems. Sensitivity, scalability, robustness, and convergence analyses were used and combined with the Wilcoxon rank-sum test and Friedman test. The simulation results show that TSO performs better compared to other comparative algorithms.

## 1. Introduction

Real-world optimization problems have become more challenging, which requires more efficient solution methods. Different scholars have studied various approaches to solve these complex and difficult problems from the real world. A part of researchers solve these optimization problems using traditional methods such as quasi-Newton, conjugate gradient, and sequential quadratic programming methods. However, owing to the nonlinear, nonproductivity characteristics of most real-world optimization problems and the involvement of multiple decision variables and complex constraints, these traditional algorithms are difficult to be solved effectively [1, 2]. The metaheuristic algorithm has the advantages of not relying on the problem model, not requiring gradient information, having strong search capability and wide applicability, and can achieve a good balance between solution quality and computational cost [3]. Therefore, the metaheuristic algorithms have been proposed to solve real-world optimization problems, such as image segmentation [4, 5], feature selection [6, 7], mission planning [8, 9], parameter optimization [10, 11], job shop scheduling [12, 13], etc.

Metaheuristic algorithms are usually classified into three categories [14]: evolution-based algorithms, physical-based algorithms, and swarm-based algorithms. The evolution-based algorithm is inspired by the laws of evolution in nature. Genetic algorithm (GA) [15], inspired by Darwin's theory of superiority and inferiority, is a well-known evolution-based algorithm. With the popularity of GA, several other widely used evolution-based algorithms have been proposed, including differential evolution (DE) [16], genetic programming (GP) [17], evolutionary strategies (ES) [18], and evolutionary programming (EP) [19]. In addition, several new evolution-based algorithms have been proposed, such as artificial algae algorithm (AAA) [20], biogeography-based optimization (BBO) [21], and monkey king evolutionary (MKE) [22]. The physical-based algorithms are inspired by various laws of physics. One of the most famous algorithms of this category is simulated annealing (SA) [23]. SA is inspired by the law of thermodynamics in which a material is heated up and then cooled slowly. There are other physical-based algorithms proposed, including gravitational search algorithm (GSA) [24], nuclear reaction optimization (NRO) [25], water cycle algorithm (WCA) [26], and sine cosine algorithm (SCA) [27]. The swarm-based algorithms are inspired by the social behavior of different species in natural groups. Particle swarm optimization (PSO) [28] and ant colony optimization (ACO) [29] are two typical swarm-based algorithms. PSO and ACO mimic the aggregation behavior of bird colonies and the foraging behavior of ant colonies, respectively. Some other algorithms of this category include: grey wolf optimizer (GWO) [30], monarch butterfly optimization (MBO) [31], elephant herding optimization (EHO) [32], moth search algorithm (MSA) [33], manta ray foraging optimization (MRFO) [34],earthworm optimization algorithm (EOA) [35], etc. With the development of metaheuristics, a type of human-based metaheuristic algorithm is also emerging. These algorithms are inspired by the characteristics of human activity. Teaching-learning-based optimization (TLBO) [36], inspired by traditional teaching methods, is a typical example of this category among metaheuristic algorithms. Other human-based metaheuristics include: social evolution and learning optimization (SELO) [37], group teaching optimization algorithm (GTOA) [38], heap-based optimizer (HBO) [39], political optimizer (PO) [40], etc.

There is a common feature of all these metaheuristic algorithms that rely on exploration and exploitation in the search space to find the optimal solution [41, 42]. Exploration means that the algorithm searches for promising regions in a wide search space and exploitation is a further search for the best solution in the promising regions. The balance of the two search behaviors affects the quality of the solution. When exploration dominates, exploitation declines, and vice versa. Therefore, it is a big challenge to balance exploration and exploitation for metaheuristics. Although there are constantly new algorithms being developed, the no free lunch (NFL) [43] theory states that no particular algorithm can solve all optimization problems perfectly. The NFL has motivated researchers to develop effective metaheuristic algorithms to solve various fields of optimization problems.

In this paper, a novel swarm-based metaheuristic is presented called tuna swarm optimization (TSO). It is inspired by two types of swarm foraging behavior of tunas. The TSO is evaluated in 23 benchmark functions and 3 engineering design problems. Test results reveal that the method proposed in this paper significantly outperforms those popular and recent metaheuristics. This paper is structured as follows: Section 2 describes the inspiration for TSO and builds the corresponding mathematical model. A benchmark function set and three engineering design problems are employed to check the performance of TSO in Sections 3 and 4, respectively.  Section 5 concludes the overall work and provides an outlook for the future.

## 2. Tuna Swarm Optimization

### 2.1. Inspiration

Tuna, scientifically named Thunnini, is a marine carnivorous fish. There are many species of tuna, and the size varies greatly. Tuna are top marine predators, feeding on a variety of midwater and surface fish. Tunas are continuous swimmers, and they have a unique and efficient way of swimming (called fishtail shape) in which the body stays rigid while the long, thin tail swings rapidly. Although the single tuna swims very fast, it is still not as fast as the nimble small fish response. Therefore, the tuna will use the “ group travel “ method for predation. They use their intelligence to find and attack their prey. These creatures have evolved a variety of effective and intelligent foraging strategies.

The first strategy is spiral foraging. When tuna are feeding, they swim by forming a spiral formation to drive their prey into shallow water where they can be attacked more easily.

The second strategy is parabolic foraging. Each tuna swims after the previous individual, forming a parabolic shape to enclose its prey.

Tuna successfully forage by the above two methods. In this paper, a new swarm-based metaheuristic optimization algorithm, namely, tuna swarm optimization, is proposed based on modeling these natural foraging behaviors.

### 2.2. Mathematical Model

In this section, the mathematical model of the proposed algorithm is described in detail.

#### 2.2.1. Initialization

Similar to most swarm-based metaheuristics, TSO starts the process of optimization by generating initial populations at random uniformly in the search space,

(1) $$\begin{array}{c} X_{i}^{\mathrm{int}}=rand\cdot \left(ub-lb\right)+lb,\;i=1,2,...,NP, \end{array}$$

where **X**_*i*_^int^ is the *i*^*th*^ initial individual, **ub** and **lb** are the upper and lower boundaries of the search space, *NP* is the number of tuna populations, and **rand** is a uniformly distributed random vector ranging from 0 to 1.

#### 2.2.2. Spiral Foraging

When sardines, herring, and other small schooling fish encounter predators, the entire school of fish forms a dense formation constantly changing the swimming direction, making it difficult for predators to lock a target. At this time, the tuna group chase the prey by forming a tight spiral formation. Although most of the fish in the school have little sense of direction, when a small group of fish swim firmly in a certain direction, the nearby fish will adjust their direction one after another and finally form a large group with the same goal and start to hunt. In addition to spiraling after their prey, schools of tuna also exchange information with each other. Each tuna follows the previous fish, thus enabling information sharing among neighboring tuna. Based on the above principles, the mathematical formula for the spiral foraging strategy is as follows:

(2) $$\begin{array}{c} X_{i}^{t+1}=\left\{\begin{array}{c} \alpha_{1}\cdot \left(X_{best}^{t}+\beta \cdot \left|X_{best}^{t}-X_{i}^{t}\right|\right)+\alpha_{2}\cdot X_{i}^{t},\;i=1,\\ \alpha_{1}\cdot \left(X_{best}^{t}+\beta \cdot \left|X_{best}^{t}-X_{i}^{t}\right|\right)+\alpha_{2}\cdot X_{i-1}^{t},\;i=2,3,...,NP, \end{array}\right. \end{array}$$

(3) $$\begin{array}{c} \alpha_{1}=a+\left(1-a\right)\cdot \frac{t}{t_{\max}}, \end{array}$$

(4) $$\begin{array}{c} \alpha_{2}=\left(1-a\right)-\left(1-a\right)\cdot \frac{t}{t_{\max}}, \end{array}$$

(5) $$\begin{array}{c} \beta =e^{bl}\cdot \cos\left(2\pi b\right), \end{array}$$

(6) $$\begin{array}{c} l=e^{3\cos\left(\left(\left(t_{\max}+1/t\right)-1\right)\pi \right)}, \end{array}$$

where **X**_*i*_^*t*+1^ is the *i*^*th*^ individual of the *t*+1 iteration, **X**_*best*_^*t*^ is the current optimal individual (food), *α*_1_ and *α*_2_ are weight coefficients that control the tendency of individuals to move towards the optimal individual and the previous individual, *a* is a constant used to determine the extent to which the tuna follow the optimal individual and the previous individual in the initial phase, *t* denotes the number of current iteration, *t*_max_ is the maximum iterations, and *b* is a random number uniformly distributed between 0 and 1.

When all tuna forage spirally around the food, they have good exploitation ability for the search space around the food. However, when the optimal individual fails to find food, blindly following the optimal individual to forage is not conducive to group foraging. Therefore, we consider generating a random coordinate in the search space as a reference point for spiral search. This facilitates each individual to search a wider space and makes TSO with global exploration ability. The specific mathematical model is described as follows:

(7) $$\begin{array}{c} X_{i}^{t+1}=\left\{\begin{array}{c} \alpha_{1}\cdot \left(X_{rand}^{t}+\beta \cdot \left|X_{rand}^{t}-X_{i}^{t}\right|\right)+\alpha_{2}\cdot X_{i}^{t},\;i=1,\\ \alpha_{1}\cdot \left(X_{rand}^{t}+\beta \cdot \left|X_{rand}^{t}-X_{i}^{t}\right|\right)+\alpha_{2}\cdot X_{i-1}^{t},\;i=2,3,...,NP, \end{array}\right. \end{array}$$

where **X**_*rand*_^*t*^ is a randomly generated reference point in the search space.

In particular, metaheuristic algorithms usually perform extensive global exploration in the early stage and then gradually transition to precise local exploitation. Therefore, TSO changes the reference points of spiral foraging from random individuals to optimal individuals as the iteration increases. In summary, the final mathematical model of the spiral foraging strategy is as follows:

(8) $$\begin{array}{c} X_{i}^{t+1}=\left\{\begin{array}{c} \begin{array}{c} \alpha_{1}\cdot \left(X_{rand}^{t}+\beta \cdot \left|X_{rand}^{t}-X_{i}^{t}\right|\right)+\alpha_{2}\cdot X_{i}^{t},\;i=1,\\ \alpha_{1}\cdot \left(X_{rand}^{t}+\beta \cdot \left|X_{rand}^{t}-X_{i}^{t}\right|\right)+\alpha_{2}\cdot X_{i-1}^{t},\;i=2,3,...,NP, \end{array},\text{ if rand}<\frac{t}{t_{\max}},\\ \begin{array}{c} \alpha_{1}\cdot \left(X_{best}^{t}+\beta \cdot \left|X_{best}^{t}-X_{i}^{t}\right|\right)+\alpha_{2}\cdot X_{i}^{t},\;i=1,\\ \alpha_{1}\cdot \left(X_{best}^{t}+\beta \cdot \left|X_{best}^{t}-X_{i}^{t}\right|\right)+\alpha_{2}\cdot X_{i-1}^{t},\;i=2,3,...,NP, \end{array},\text{ if rand}\geq \frac{t}{t_{\max}}. \end{array}\right. \end{array}$$

#### 2.2.3. Parabolic Foraging

In addition to feeding by forming a spiral formation, tunas also form a parabolic cooperative feeding. Tuna forms a parabolic formation with food as a reference point. In addition, tuna hunt for food by searching around themselves. These two approaches are performed simultaneously, with the assumption that the selection probability is 50% for both. The specific mathematical model is described as follows:

(9) $$\begin{array}{c} X_{i}^{t+1}=\left\{\begin{array}{c} X_{best}^{t}+rand\cdot \left(X_{best}^{t}-X_{i}^{t}\right)+TF\cdot p^{2}\cdot \left(X_{best}^{t}-X_{i}^{t}\right),\;\text{if rand }<0.5\text{,}\\ TF\cdot p^{2}\cdot X_{i}^{t},\;\text{if rand }\geq \text{0.}5\text{,} \end{array}\right. \end{array}$$

(10) $$\begin{array}{c} p={\left(1-\frac{t}{t_{\max}}\right)}^{\left(t/t_{\max}\right)}, \end{array}$$

where *TF* is a random number with a value of 1 or −1.

**Figure 1:**
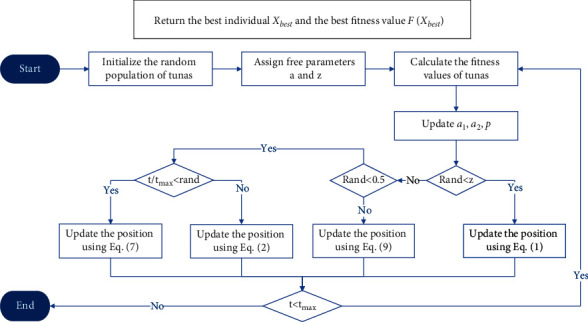
Flowchart of TSO.

Tuna hunt cooperatively through two foraging strategies and then find their prey. For the optimization process of TSO, the population is first randomly generated in the search space. In each iteration, each individual randomly chooses one of the two foraging strategies to execute, or chooses to regenerate the position in the search space according to probability *z*. The value of parameter *z* will be discussed in the parameter setting simulation experiments. During the entire optimization process, all individuals of TSO are continuously updated and calculated until the end condition is met, and then the optimal individual and the corresponding fitness value are returned. The TSO pseudocode is shown in Algorithm 1. The detailed process of TSO is shown in Figure 1.

## 3. Numerical Experiment and Discussion

### 3.1. Benchmark Function Set and Compared Algorithms

**Figure 2:**
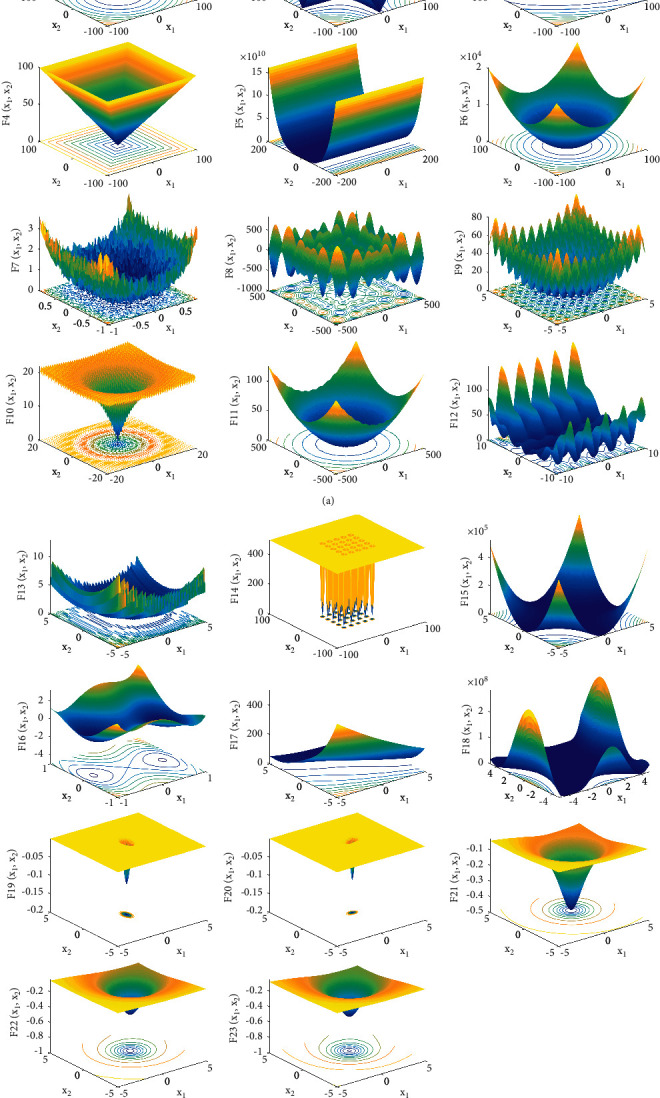
3D visualization for 2D benchmark functions.

In this section, in order to evaluate the performance of the TSO proposed in this paper, a set of well-known benchmark functions are employed for testing. This set of benchmark functions include 7 unimodal functions, 6 multimodal functions, and 10 multimodal functions with fixed dimensions. The unimodal functions F1–F7 have only one global optimal solution and are, therefore, often employed to evaluate the local exploitation capability of an algorithm. Besides the global optimal solution, the multimodal functions F8–F23 also have multiple local optimal solutions and are, therefore, used to challenge the global exploration capability and local optimal avoidance capability of an algorithm. The mathematical formulas and characteristics of these functions are shown in Table 1. A three-dimensional visualization of these functions is given in Figure 2.

### 3.2. Compared Algorithms and Experimental Setup

The results of the proposed TSO are compared with seven well-regarded and recent metaheuristics. These algorithms include particle swarm optimization (PSO) [28], grey wolf optimizer (GWO) [30], whale optimization algorithm (WOA) [44], and Salp swarm algorithm (SSA) [45], which are the more frequently used algorithms in the optimization field, and Harris Hawks optimization (HHO) [46], equilibrium optimizer (EO) [47], and tunicate swarm algorithm (TSA) [48], which are three new algorithms recently proposed.

All algorithms were implemented under MATLAB R2016b on a computer with Windows 10 64 bit Professional and 16 GB RAM. The population size and the maximum number of iterations for all optimizers were set to 50 and 1000, respectively. All results were recorded and compared based on the performance of each optimizer on 30 independent runs. It is well known that the parameter settings of an algorithm have a huge impact on the performance of the algorithm. For the fair comparison, the parameter settings of all compared algorithms are based on the parameters used by the authors of the original article.  Table 2 lists the parameters used by each algorithm.

### 3.3. Analysis of TSO for Variable-Dimensional Functions

Table 3 presents the results of TSO and other comparison algorithms for solving F1–F13 with Dim = 30. In addition, the performance of TSO is also evaluated using test functions with different dimensions, which is beneficial to recognize the ability of TSO to solve high-dimensional functions.  Table 4–Table 6 show the results of TSO and other comparison algorithms for processing F1–F13 with dimensions of 100, 500, and 1000.

**Figure 3:**
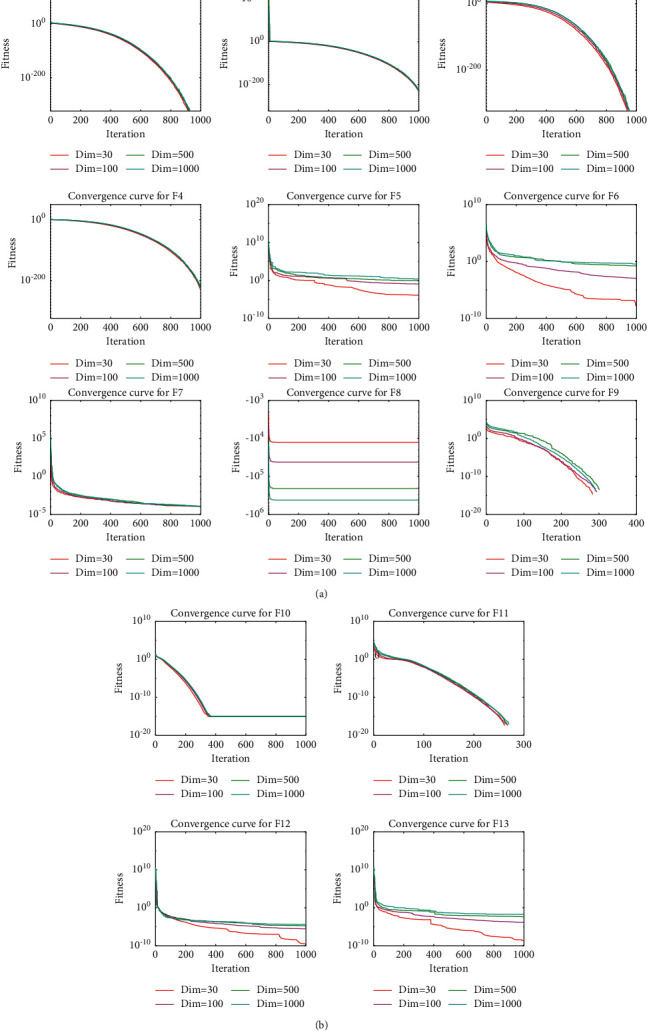
Convergence curves of TSO on 13 test functions in 4 different dimensional cases.

As shown in the results of the unimodal functions F1–F7 in Table 3–Table 6, TSO achieves the best results in most of the functions, significantly outperforming almost all the comparison algorithms. In addition, TSO outperforms the comparison algorithms still when dealing with high-dimensional problems. On the other hand, the results obtained by TSO are not much fluctuated as the dimensionality increases, which can also be observed in the convergence curves in Figure 3. Specifically, TSO performs best on F1–F5 when Dim = 30. In particular, TSO can consistently obtain the theoretical optimal solution on F1 and F3. In F7, HHO is the best optimizer, and TSO follows the best. The TSO performs poorly for the F6. For high-dimensional functions, TSO and HHO perform in the top 2. TSO gives the most satisfactory results for F1–F4. HHO performs best on F5–F7, with TSO ranking behind it. Overall, TSO performs the best exploitation ability among all the algorithms involved in the test for unimodal functions on different dimensions.

The results for solving the multimodal functions F8–F13 in different dimensions for each algorithm are also given in Table 3–Table 6. The analysis shows that TSO performs best in all dimensions when solving F8–F11. The TSO ranks behind the HHO in solving F12 and F13. Notably, TSO can stably obtain the theoretical optimal solution for F9–F11. As the convergence curves show, the TSO performance does not degrade too much as the dimensionality increases, showing the superior performance of TSO in solving high-dimensional multimodal functions.

### 3.4. Analysis of TSO for Fixed Dimensional Functions

**Figure 4:**
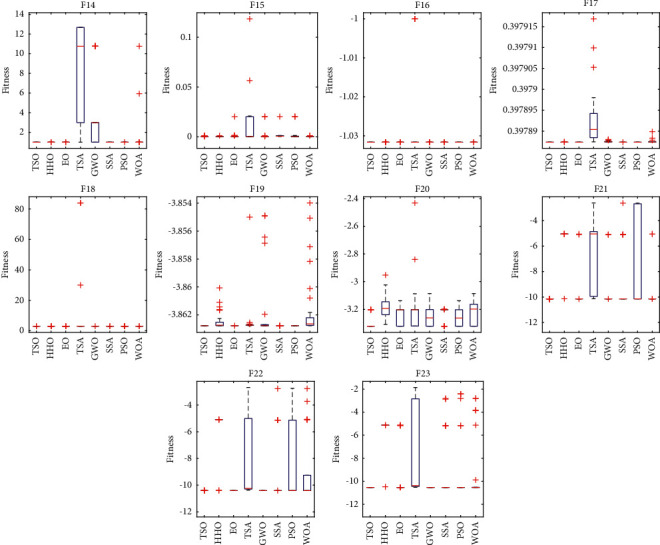
Boxplot analysis for fixed dimensional functions.

The test results of TSO applied to fixed dimensional functions are shown in 7. The means in the table show that TSO is superiorly competitive on the fixed dimensional functions, performing best on eight of the ten functions. TSO ranks second and third on F8 and F15. In order to analyze the distribution characteristics of TSO when solving fixed dimensional functions, box plots of F14–F23 are drawn based on the results of 30 runs, as shown in Figure 4. It can be observed that TSO outperforms the comparison algorithm in most functions in terms of maximum, minimum and median values, and the distribution of solutions is more concentrated, thus, TSO performs better compared to other algorithms.

### 3.5. Wall-Clock Time Analysis of TSO

**Figure 5:**
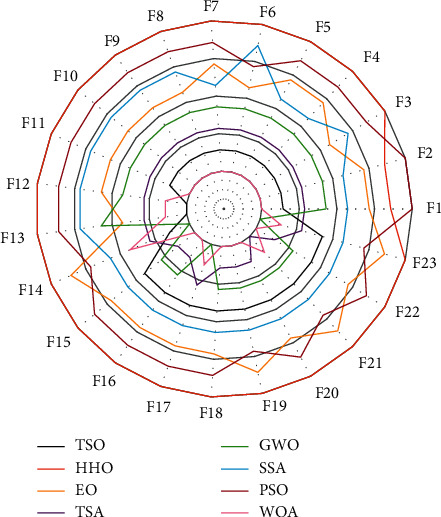
Time cost ranking result.

Computational efficiency is also an important measure of algorithm performance.  Table 8 records the average computational time consumed by these algorithms for 30 independent runs in each function. It can be seen that the computation of TSO does not take much time, only longer than WOA and TSA. Although TSO takes more time, the performance is better than WOA and TSA. Moreover, TSO takes less time with better performance than other comparison algorithms, thus TSO has a huge efficiency advantage.  Figure 5 illustrates the ranking of computational time consumption of each algorithm, and it can be visually seen that WOA, TSA, and TSO rank in the top three.

### 3.6. Parameter Sensitivity Analysis

This section focuses on the analysis of the values of the two control parameters (z and a) of the TSO. The first parameter is *z*, which controls the probability of randomly generated individuals. The second parameter is *a*, which controls the extent to which each individual follows the optimal individual and the neighboring individuals. The 13 variable dimensional functions (F1–F13) and 10 fixed dimensional functions (F14–F23) are used for analyzing the effect of the values of the control parameters on the TSO performance. The values of each parameter are defined as *a*={0.1, 0.2, 0.3, ..., 0.9}, *z*={0,0.01, 0.02, ..., 0.09, 0.1}, and there are 9 × 11=99 combinations in total. Each combination solves the test functions 30 times independently, and a total of 68310 data are obtained. Due to the large amount of data, no specific comparison of the experimental results was performed, but the differences in the experimental results were reflected by sorting the simulation results under different parameter settings by Friedman's test.

According to the results, the Friedman test results of unimodal functions F1–F7, multimodal functions F8–F23, and all functions F1–F23 are given respectively, as shown in Table 9–Table 11. From the results in Table 9, it is clear that the smaller the value of *z* taken, the better the TSO performance. The larger the value of *a* taken, the better the results obtained by TSO. This is because the smaller the value of*z*is, the smaller the probability of randomly generating new individuals, while the larger the value of *a* is, the higher the degree to which each individual follows the optimal individual, all of which are beneficial for improving exploitation ability and accelerating convergence. For the multipeaked functions F14–F23, we can get almost the opposite conclusion from Table 10 compared to unimodal functions. The rankings considering the results of all functions are given in Table 11. The results show that TSO has the best performance when *z*=0.05, *a*=0.7.

### 3.7. Statistical Analysis of TSO

This section further analyses the differences between TSO and other algorithms statistically using the Wilcoxon rank-sum test and Friedman test. The Wilcoxon rank-sum test is a paired test that checks for significant differences between two algorithms. The results of the test between TSO and each algorithm at significance level *α*=0.05 are given in Table 12–Table 16, where the symbols “+/ = /-” indicate that TSO performs better, similar, or worse than the comparison algorithm.  Table 17 gives the statistical results of TSO in different dimensions and functions that are better than, similar to, and worse than the comparison algorithm. TSO outperforms other comparative algorithms in different cases and achieves results of 32/15/15, 42/13/7, 62/0/0, 61/1/0, 61/1/0, 51/6/5,and 55/7/0, confirming the significant superiority of MSMA in most cases compared to other algorithms.

Table 18 shows the statistics of F1–F13 in different dimensions and the fixed dimensional functions F14–F23. The statistics show that TSO ranks first in all cases. Therefore, it can be considered that TSO has the best performance compared to other algorithms.

## 4. TSO for Engineering Design Problems

This section uses three engineering design problems to assess TSO's ability to solve real-world problems. These problems include the pressure vessel design problem, the tension/compression spring design problem, and the welded beam design problem. TSO uses the same number of iterations (1000) and populations (50) in solving these engineering design problems. Each problem is run 30 times independently, and the statistical results are compared with other algorithms in the literature.

### 4.1. Pressure Vessel Design

**Figure 6:**
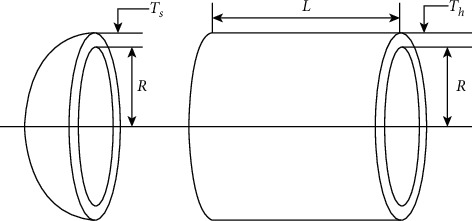
Schematic of the pressure vessel design problem (Figure 6 is reproduced from [49]).

The pressure vessel design problem shown in Figure 6 is a well-known benchmark test design problem with the goal of reducing total cost, including forming cost, material cost, and welding cost. There are four different variables: vessel thickness Ts (*x*_1_), head thickness Th (*x*_2_), inner diameter *R* (*x*_3_), and vessel cylindrical cross-section length *L* (*x*_4_). The problem is described as follows:

(11) $$\begin{array}{c} \min f\left(x_{1},x_{2},x_{3},x_{4}\right)=0.6224x_{1}x_{3}x_{4}+1.7781x_{2}x_{3}^{2}+3.1661x_{1}^{2}x_{4}+19.84x_{1}^{2}x_{3}. \end{array}$$

Subject to

(12) $$\begin{array}{c} g_{1}\left(X\right)=-x_{1}+0.0193x_{3}\leq 0,\\ g_{2}\left(X\right)=-x_{2}+0.00954x_{3}\leq 0,\\ g_{3}\left(X\right)=-\pi x_{3}^{2}x_{4}-\frac{4}{3}\pi x_{3}^{2}+1,296,000\leq 0,\\ g_{4}\left(X\right)=x_{4}-240\leq 0,\\ Variable\;ranges:\;1\times 0.0625\leq x_{1},\\ x_{2}\leq 99\times 0.0625,\;10\leq x_{3},\;x_{4}\leq 200. \end{array}$$

The results of TSO for solving this problem are compared with other algorithms such as DDSCA, ISCA, MBA, CPSO, TEO, hHHO-SCA, HPSO, MVO, and AFA, and the comparison is shown in Table 19. The results show that the TSO solution is superior to the solutions provided by the comparison algorithms with optimal solutions for each parameter [0.7782, 0.3846, 40.3196, and 199.9999], corresponding to a minimum cost of 5885.3327.

### 4.2. Tension/Compression Spring Design

**Figure 7:**
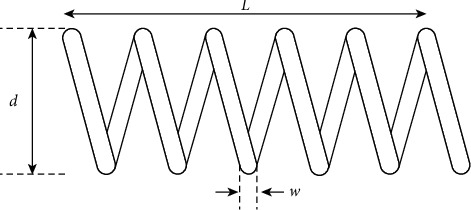
Schematic of tension/compression spring design problem.

The tension/compression spring design problem is a mechanical engineering design optimization problem. As shown in Figure 7, the goal of this problem is to reduce the weight of the spring. It includes four nonlinear inequalities and three continuous variables: wire diameter w(*x*_1_), average coil diameter d(*x*_2_), and coil length or number L(*x*_3_). This problem can be described by the following equation:

(13) $$\begin{array}{c} \min f\left(x_{1},x_{2},x_{3}\right)=\left(x_{3}+2\right)x_{1}^{2}x_{2}. \end{array}$$

Subject to

(14) $$\begin{array}{c} g_{1}\left(X\right)=1-\frac{x_{2}^{3}x_{3}}{71785x_{1}^{4}}\leq 0,\\ g_{2}\left(X\right)=\frac{x_{2}\left(4x_{2}-x_{1}\right)}{12566x_{1}^{3}\left(x_{2}-x_{1}\right)}+\frac{1}{5108x_{1}^{2}}-1\leq 0,\\ g_{3}\left(X\right)=1-\frac{140.45x_{1}}{x_{2}^{2}x_{3}}\leq 0,\\ g_{4}\left(X\right)=\frac{2\left(x_{1}+x_{2}\right)}{3}-1\leq 0,\\ Variable\;range:\;0.05\leq x_{1}\leq 2,\;0.25\leq x_{2}\leq 1.3,\\ 2.0\leq x_{3}\leq 15.0. \end{array}$$

The solution of TSO is compared with other methods given in the literature, including GA3, CPSO, CDE, DDSCA, GSA, hHHO-SCA, AEO, and MVO.  Table 20 shows the parameters and costs corresponding to the optimal solution of each algorithm. As can be seen from Table 10, TSO is the best algorithm for solving the problem. The optimal solution for each parameter corresponding to the lowest cost of 1.724852 is [0.205729, 3.470488, 9.036623, 0.205729].

### 4.3. Welded Beam Design

**Figure 8:**
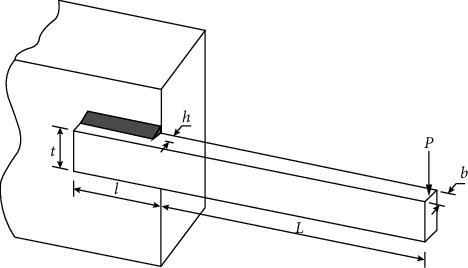
Schematic of the welded beam design problem.

The welded beam design problem is the classical structural optimization problem. As shown in Figure 8, the objective of this design problem is to minimize the fabrication cost of the welded beam. The optimization variables include welding thickness h(*x*_1_), joint beam length l(*x*_2_), beam height *t*(*x*_3_), and beam thickness b(*x*_4_). The mathematical model is as follows:

(15) $$\begin{array}{c} \min f\left(x_{1},x_{2},x_{3},x_{4}\right)=1.10471x_{1}^{2}x_{2}+0.04811x_{3}x_{4}\left(14.0+x_{2}\right). \end{array}$$

Subject to

(16) $$\begin{array}{c} g_{1}\left(X\right)=\tau_{d}-\tau \left(X\right)\geq 0,\\ g_{2}\left(X\right)=\sigma_{d}-\sigma \left(X\right)\geq 0,\\ g_{3}\left(X\right)=x_{4}-x_{1}\geq 0,\\ g_{4}\left(X\right)=P_{c}\left(X\right)-P\geq 0,\\ g_{5}\left(X\right)=\delta_{d}-\delta \left(X\right)\geq 0, \end{array}$$

where

(17) $$\begin{array}{c} \tau \left(X\right)=\sqrt{\frac{{\left(\tau^{\prime }\left(X\right)\right)}^{2}+{\left(\tau^{\prime \prime }\left(X\right)\right)}^{2}+x_{2}\tau^{\prime }\left(X\right)\tau^{\prime \prime }\left(X\right)}{\sqrt{0.25\left(x_{2}^{2}+{\left(x_{1}+x_{3}\right)}^{2}\right)}}},\\ \sigma \left(X\right)=\frac{50400}{x_{3}^{2}x_{4}},\\ P_{c}\left(X\right)=64746.002\left(1-0.0282346x_{3}\right)x_{3}x_{4}^{3},\\ \delta \left(X\right)=\frac{2.1952}{x_{3}^{2}x_{4}},\\ \tau^{\prime }\left(X\right)=\frac{6000}{\left(\sqrt{2}x_{1}x_{2}\right)},\\ \tau^{\prime \prime }\left(X\right)=\frac{6000\left(14+0.5x_{2}\right)\sqrt{0.25\left(x_{2}^{2}+{\left(x_{1}+x_{3}\right)}^{2}\right)}}{2\left(0.707x_{1}x_{2}\left(x_{2}^{2}/12+0.25{\left(x_{1}+x_{3}\right)}^{2}\right)\right)}. \end{array}$$

This problem has been solved by different algorithms such as DDSCA, HGA, MGWO-III, IAPSO, TEO, hHHO-SCA, HPSO, CPSO, and WCA.  Table 21 summarizes the results of the above algorithms and compares them with the best results of TSO. The results show that TSO can provide a parameter design plan with lower cost compared to other algorithms. TSO generates the best solution at design variables of 0.205729, 3.470490, 9.036626, and 0.205729 with a minimum cost of 1.724854.

## 5. Conclusions

This work presents a novel swarm-based metaheuristic algorithm: tuna swarm optimization. The algorithm is inspired by the cooperative foraging mechanisms of tuna, including spiral foraging and parabolic foraging. The method has few adjustable parameters and can be implemented easily. TSO was comprehensively evaluated using a set of benchmark functions in different dimensions and was compared with other state-of-the-art algorithms. The results show that TSO is superior to the comparative algorithms. In addition, the pressure vessel design problem, the tension/compression spring design problem, and the welded beam design problem are investigated. The statistical results show that TSO has a high potential for solving real-world optimization problems compared to the reported methods. A major factor in TSO's success is the balance of exploitation and exploration achieved through the two foraging strategies. Meanwhile, fewer iterative steps bring less time costs, which is one of the strengths of TSO. However, while TSO performs excellently in most functions, there is still potential for enhancement regarding the small percentage of functions. This can be done by further enhancing TSO's ability to get rid of local optimum, using methods such as hybridisation of algorithms, adaptive parameters, etc.

For future work, binary and multiobjective versions of TSO can be developed for discrete problems and multiobjective optimization problems. Moreover, TSO will be applied to solve UAV mission planning problems such as trajectory planning problems, target allocation problems, etc. A further interesting direction would be to investigate the performance of different constraint handling methods in solving constrained optimization problems.

## Figures and Tables

**Algorithm 1:**
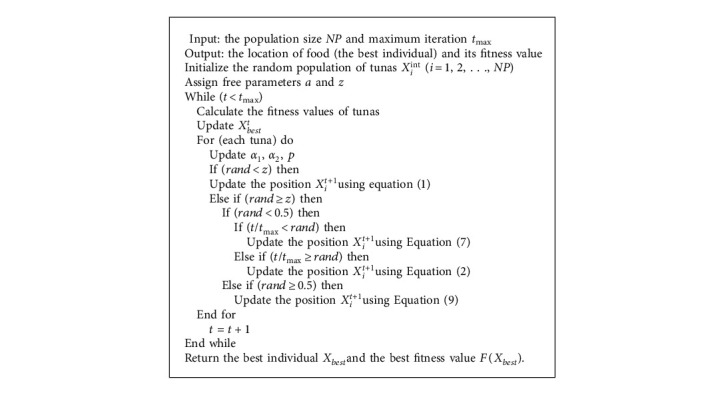
Pseudocode of TSO.

**Table 1:** Description of benchmark functions.

Test function	Name	Type	*Dim*	Range	Optimum
*f*_01_(*x*)=∑_*i*=1_^*D*^*x*_*i*_^2^	Sphere	US	30	[−100, 100]	0
*f*_02_(*x*)=∑_*i*=1_^*D*^|*x*_*i*_|+∏_*i*−1_^*D*^|*x*_*i*_|	Schwefel 2.22	UN	30	[−10, 10]	0
*f*_03_(*x*)=∑_*i*=1_^*D*^(∑_*j*−1_^*D*^*x*_*i*_)^2^	Schwefel 1.2	UN	30	[−100, 100]	0
*f*_04_(*x*)=max_*i*_{|*x*_*i*_|, 1 ≤ *i* ≤ *D*}	Schwefel 2.21	US	30	[−100, 100]	0
*f*_05_(*x*)=∑_*i*=1_^*D*^100(*x*_*i*+1_^2^ − *x*_*i*_^2^)^2^+(*x*_*i*_ − 1)^2^	Rosenbrock	UN	30	[−30, 30]	0
*f*_06_(*x*)=∑_*i*=1_^*D*^(⌊*x*_*i*_+0.5⌋)^2^	Step	US	30	[−100, 100]	0
*f*_07_(*x*)=∑_*i*=1_^*D*^*ix*_*i*_^4^+*random*[0,1)	Quartic	US	30	[−1.28, 1.28]	0

$f_{08}\left(x\right)=\sum_{i=1}^{D}-x_{i}\sin\left(\sqrt{\left|x_{i}\right|}\right)$	Schwefel 2.26	MS	30	[−500, 500]	−418.9829^*∗*^D
*f*_09_(*x*)=∑_*i*=1_^*D*^(*x*_*i*_^2^ − 10cos(2*πx*_*i*_)+10sin)	Rastrigin	MS	30	[−5.12, 5.12]	0
$f_{10}\left(x\right)=20+e-20\exp\left(-20\sqrt{\left(1/D\right)\sum_{i=1}^{D}x_{i}^{2}}\right)-\exp\left(\left(1/D\right)\sum_{i=1}^{D}\cos\left(2\pi x_{i}\right)\right)$	Ackley	MS	30	[−32, 32]	8.8818e−16
$f_{11}\left(x\right)=\left(1/4000\right)\sum_{i=1}^{D}\left(x_{i}^{2}\right)-\left(\prod_{i=1}^{D}\cos\left(x_{i}/\sqrt{i}\right)\right)+1$	Griewank	MN	30	[−600, 600]	0
$f_{12}\left(x\right)=\left(\pi /D\right)\left\{10\sin^{2}\left(\pi y_{i}\right)+\sum_{i=1}^{D}{\left(y_{i}-1\right)}^{2}\left[1+10\sin^{2}\left(\pi y_{i+1}\right)\right]+\left(y_{D}-1\right)\right\}+\sum_{i=1}^{D}u\left(x_{i},10,100,4\right),\;y_{i}=1+\left(\left(x_{i}+1\right)/4\right)u\left(x_{i},a,,km\right)=\left\{\begin{array}{c} k{\left(x_{i}-a\right)}^{m},\;x_{i}>a\\ 0,\;-a<x_{i}<a\\ k{\left(-x_{i}-a\right)}^{m},\;x_{i}<a \end{array}\right.$	Penalized	MN	30	[−50, 50]	0
*f*_13_(*x*)=0,1{sin^2^(3*πx*_*i*_)+∑_*i*=1_^*D*^(*x*_*i*_ − 1)^2^[1+sin^2^(3*πx*_*i*_)]+(*x*_*D*_ − 1)^2^[1+sin^2^(2*πx*_*D*_)]}+∑_*i*−1_^*D*^*u*(*x*_*i*_, 5,100,4)(*π*/*D*){10sin^2^(*πy*_*i*_)+}+∑_*i*=1_^*D*^*u*(*x*_*i*_, 10,100,4)	Penalized2	MN	30	[−50, 50]	0

*f*_14_(*x*)=((1/500)+∑_*j*=1_^25^(1/(*j*+∑_*i*=1_^2^(*x*_*i*_ − *a*_*ij*_)^6^)))^−1^	Foxholes	MS	2	[−65.53, 65.53]	0.998004
*f*_15_(*x*)=∑_*i*=1_^11^(*a*_*i*_ − (*x*_1_(*b*_*i*_^2^+*b*_*i*_*x*_2_)/*b*_*i*_^2^+*b*_*i*_*x*_3_+*x*_4_))^−1^	Kowalik	MS	4	[−5, 5]	0.0003075
*f*_16_(*x*)=(4*x*_1_^2^ − 2.1*x*_1_^4^+1/3*x*_1_^6^+*x*_1_*x*_2_ − 4*x*_2_^2^+*x*_2_^4^)	Six hump camel back	MN	2	[−5, 5]	−1.03163
*f*_17_(*x*)=(*x*_2_ − (5.1/4*π*^2^)*x*_1_^2^+(5/*π*)*x*_1_ − 6)^2^+10(1 − (1/8*π*)cos*x*_1_+10)	Branin	MS	2	[−5, 10]×[0, 15]	0.398
*f*_18_(*x*)=[1+(*x*_1_+*x*_2_+1)^2^(19 − 14*x*_1_+3*x*_1_^2^ − 14*x*_2_+6*x*_1_*x*_2_+3*x*_2_^2^)] × [30+(2*x*_1_ − 3*x*_2_)^2^(18 − 32*x*_1_+12*x*_1_^2^+48*x*_2_ − 36*x*_1_*x*_2_+27*x*_2_^2^)]	Goldstein price	MN	2	[−5, 5]	3
*f*_19_(*x*)=−∑_*i*=1_^4^(*c*_*i*_exp(−∑_*j*−1_^3^*a*_*ij*_(*x*_*i*_ − *p*_*ij*_))^2^)	Hartman 3	MN	3	[0, 1]	−3.8628
*f*_20_(*x*)=−∑_*i*=1_^4^(*c*_*i*_exp(−∑_*j*−1_^6^*a*_*ij*_(*x*_*i*_ − *p*_*ij*_))^2^)	Hartman 6	MN	6	[0, 1]	−3.32
*f*_21_(*x*)=−∑_*i*=1_^5^[(*X* − *a*_*i*_)(*X* − *a*_*i*_)^*T*^+*c*_*i*_]^−1^	Langermann 5	MN	4	[0, 10]	−10.1532
*f*_22_(*x*)=−∑_*i*=1_^7^[(*X* − *a*_*i*_)(*X* − *a*_*i*_)^*T*^+*c*_*i*_]^−1^	Langermann 7	MN	4	[0, 10]	−10.4029
*f*_23_(*x*)=−∑_*i*=1_^10^[(*X* − *a*_*i*_)(*X* − *a*_*i*_)^*T*^+*c*_*i*_]^−1^	Langermann 10	MN	4	[0, 10]	−10.5364

**Table 2:** Parameter settings for algorithms.

Algorithm	Parameters
PSO	*c*_1_=*c*_2_=2, *wMax*=0.9, *wMin*=0.2
GWO	*a*=2 (linearly decreased over iterations)
WOA	*a*_1_=2 (linearly decreased over iterations)
SSA	*c*_1_=*rand*, *c*_2_=*rand*
HHO	∼
EO	*a*_1_=2, *a*_2_=1
TSA	*P*_max_=4, *P*_min_=1
TSO	*z*=0.05, *a*=0.7

**Table 3:** Comparison of results on F1–F13 with 30D.

Function		TSO	HHO	EO	TSA	GWO	SSA	PSO	WOA
F1	Ave	0.00 E+00	3.92E−193	5.36E−102	2.59E−52	4.76E−70	9.09E−09	1.78E−09	4.37E−172
	Std	0.00 E+00	0.00 E+00	7.54E−102	4.91E−52	1.16E−69	1.37E−09	8.10E−09	0.00 E+00

F2	Ave	1.47E−235	3.54E−102	1.41E−57	1.15E−30	3.93E−41	4.75E−01	1.33 E+00	1.13E−108
	Std	0.00 E+00	1.17E−101	1.70E−57	4.74E−30	3.90E−41	6.71E−01	4.34 E+00	4.69E−108

F3	Ave	0.00 E+00	1.98E−155	4.95E−27	1.52E−15	5.97E−20	4.01 E+01	1.02 E+03	1.31 E+04
	Std	0.00 E+00	1.09E−154	2.01E−26	7.35E−15	1.95E−19	2.60 E+01	1.89 E+03	6.96 E+03

F4	Ave	2.39E−236	3.11E−98	2.59E−25	2.91E−04	1.92E−17	4.00 E+00	2.40 E+00	2.60 E+01
	Std	0.00 E+00	1.14E−97	6.69E−25	7.90E−04	2.98E−17	2.61 E+00	7.09E−01	2.81 E+01

F5	Ave	1.22E−04	9.96E−04	2.39 E+01	2.85 E+01	2.65 E+01	8.80 E+01	3.16 E+03	2.65 E+01
	Std	3.16E−04	1.09E−03	1.51E−01	6.96E−01	6.99E−01	1.84 E+02	1.64 E+04	3.61E−01

F6	Ave	1.77E−08	9.32E−06	1.87E−13	3.59 E+00	4.09E−01	9.62E−09	1.20E−09	4.17E−03
	Std	9.08E−08	1.44E−05	6.11E−13	7.04E−01	3.04E−01	2.38E−09	3.07E−09	2.22E−03

F7	Ave	1.15E−04	3.67E−05	4.28E−04	3.00E−03	4.73E−04	5.67E−02	2.13E−02	1.18E−03
	Std	7.56E−05	3.20E−05	2.34E−04	1.34E−03	2.14E−04	2.49E−02	8.59E−03	1.33E−03

F8	Ave	−1.26 E+04	−1.26 E+04	−9.11 E+03	−6.39 E+03	−6.05 E+03	−7.61 E+03	−9.08 E+03	−1.19 E+04
	Std	1.64E−06	8.89E−02	7.24 E+02	6.83 E+02	8.07 E+02	8.70 E+02	5.44 E+02	1.24 E+03

F9	Ave	0.00 E+00	0.00 E+00	0.00 E+00	1.51 E+02	5.41E−01	4.78 E+01	4.85 E+01	0.00 E+00
	Std	0.00 E+00	0.00 E+00	0.00 E+00	3.54 E+01	2.14 E+00	1.13 E+01	1.37 E+01	0.00 E+00

F10	Ave	8.88E−16	8.88E−16	4.56E−15	1.49 E+00	1.28E−14	1.82 E+00	8.86E−02	4.56E−15
	Std	0.00 E+00	0.00 E+00	6.49E−16	1.63 E+00	2.87E−15	8.07E−01	3.40E−01	2.38E−15

F11	Ave	0.00 E+00	0.00 E+00	0.00 E+00	6.57E−03	1.40E−03	1.07E−02	1.11E−02	7.35E−03
	Std	0.00 E+00	0.00 E+00	0.00 E+00	7.05E−03	4.58E−03	1.28E−02	1.43E−02	1.91E−02

F12	Ave	3.16E−10	8.06E−07	3.46E−03	8.00 E+00	2.53E−02	3.90 E+00	3.11E−02	1.17E−03
	Std	8.13E−10	1.06E−06	1.89E−02	4.19 E+00	1.75E−02	1.97 E+00	7.28E−02	1.53E−03

F13	Ave	1.93E−09	5.48E−06	1.78E−02	2.83 E+00	2.97E−01	7.56E−03	2.93E−03	4.86E−02
	Std	4.41E−09	5.87E−06	3.38E−02	6.31E−01	1.06E−01	1.18E−02	4.94E−03	5.55E−02

**Table 4:** Comparison of results on F1–F13 with 100D.

Function		TSO	HHO	EO	TSA	GWO	SSA	PSO	WOA
F1	Ave	0.00 E+00	5.76E−190	1.08E−72	1.04E−27	3.53E−34	9.88E−03	7.10 E+02	1.15E−168
	Std	0.00 E+00	0.00 E+00	4.12E−72	2.59E−27	5.28E−34	1.10E−02	2.55 E+03	0.00 E+00

F2	Ave	1.96E−231	3.05E−100	2.45E−42	7.52E−18	7.04E−21	1.41 E+01	3.98 E+01	6.29E−103
	Std	0.00 E+00	1.30E−99	2.64E−42	1.09E−17	3.19E−21	4.47 E+00	2.40 E+01	3.45E−102

F3	Ave	0.00 E+00	2.81E−145	2.12E−06	7.99 E+02	1.67E−01	2.21 E+04	8.11 E+04	7.18 E+05
	Std	0.00 E+00	1.54E−144	5.59E−06	1.02 E+03	3.53E−01	1.05 E+04	1.91 E+04	1.23 E+05

F4	Ave	1.49E−229	1.30E−97	5.51E−11	2.86 E+01	1.38E−04	1.96 E+01	3.25 E+01	7.08 E+01
	Std	0.00 E+00	4.59E−97	1.75E−10	9.58 E+00	2.18E−04	2.29 E+00	3.62 E+00	2.99 E+01

F5	Ave	1.15E−01	3.74E−03	9.41 E+01	9.79 E+01	9.70 E+01	7.13 E+02	1.05 E+04	9.73 E+01
	Std	4.32E−01	5.74E−03	3.34E−01	8.33E−01	8.86E−01	4.63 E+02	2.31 E+04	4.64E−01

F6	Ave	1.08E−03	3.41E−05	1.43E−01	1.38 E+01	7.36 E+00	8.23E−03	1.04 E+03	5.30E−01
	Std	2.11E−03	5.76E−05	2.04E−01	9.64E−01	1.16 E+00	8.37E−03	3.06 E+03	1.70E−01

F7	Ave	1.17E−04	4.11E−05	6.99E−04	1.31E−02	1.56E−03	7.61E−01	4.21 E+00	9.37E−04
	Std	1.62E−04	6.24E−05	3.25E−04	4.63E−03	7.36E−04	1.72E−01	7.59 E+00	1.42E−03

F8	Ave	−4.19 E+04	−4.19 E+04	−2.89 E+04	−1.45 E+04	−1.67 E+04	−2.41 E+04	−2.35 E+04	−3.79 E+04
	Std	8.33E−02	3.21E−01	1.54 E+03	7.90 E+02	2.56 E+03	1.51 E+03	1.51 E+03	4.58 E+03

F9	Ave	0.00 E+00	0.00 E+00	0.00 E+00	9.20 E+02	1.49E−01	1.34 E+02	3.14 E+02	0.00 E+00
	Std	0.00 E+00	0.00 E+00	0.00 E+00	1.38 E+02	8.18E−01	3.79 E+01	5.10 E+01	0.00 E+00

F10	Ave	8.88E−16	8.88E−16	7.52E−15	5.96E−12	7.03E−14	5.15 E+00	4.61 E+00	4.44E−15
	Std	0.00 E+00	0.00 E+00	1.23E−15	3.24E−11	4.73E−15	1.25 E+00	3.08 E+00	2.64E−15

F11	Ave	0.00 E+00	0.00 E+00	0.00 E+00	2.71E−03	7.62E−04	1.25E−01	7.31 E+00	0.00 E+00
	Std	0.00 E+00	0.00 E+00	0.00 E+00	6.26E−03	2.94E−03	2.90E−02	2.28 E+01	0.00 E+00

F12	Ave	3.03E−06	3.47E−07	1.89E−03	9.22 E+00	1.93E−01	9.90 E+00	1.02 E+01	6.08E−03
	Std	8.46E−06	5.00E−07	5.68E−03	3.74 E+00	6.19E−02	2.58 E+00	4.20 E+00	3.07E−03

F13	Ave	1.44E−04	1.19E−05	2.12 E+00	1.21 E+01	5.68 E+00	1.54 E+02	4.54 E+02	6.00E−01
	Std	2.32E−04	1.74E−05	1.15 E+00	1.61 E+00	3.89E−01	1.57 E+01	4.27 E+02	3.17E−01

**Table 5:** Comparison of results on F1–F13 with 500D.

Function		TSO	HHO	EO	TSA	GWO	SSA	PSO	WOA
F1	Ave	0.00 E+00	6.83E−192	5.35E−59	4.74E−12	2.61E−14	3.20 E+04	1.32 E+05	2.99E−165
	Std	0.00 E+00	0.00 E+00	6.41E−59	4.95E−12	1.42E−14	2.21 E+03	2.05 E+04	0.00 E+00

F2	Ave	1.24E−230	4.93E−96	5.52E−35	9.54E−09	5.31E−09	3.39 E+02	9.89 E+02	1.12E−104
	Std	0.00 E+00	2.70E−95	3.87E−35	7.88E−09	1.32E−09	1.38 E+01	1.25 E+02	5.58E−104

F3	Ave	0.00 E+00	1.08E−87	4.43 E+02	9.00 E+05	8.42 E+04	5.87 E+05	2.20 E+06	2.63 E+07
	Std	0.00 E+00	5.91E−87	1.54 E+03	1.17 E+05	3.48 E+04	2.88 E+05	2.95 E+05	6.32 E+06

F4	Ave	2.22E−228	7.26E−94	8.36 E+01	9.91 E+01	5.10 E+01	3.24 E+01	7.55 E+01	7.29 E+01
	Std	0.00 E+00	3.97E−93	1.93 E+01	2.47E−01	6.00 E+00	2.31 E+00	2.98 E+00	2.37 E+01

F5	Ave	9.10E−01	1.53E−02	4.96 E+02	4.98 E+02	4.97 E+02	5.49 E+06	1.81 E+08	4.95 E+02
	Std	1.41 E+00	1.80E−02	7.74E−01	2.08E−01	4.29E−01	9.42 E+05	1.14 E+08	2.54E−01

F6	Ave	1.68E−01	1.01E−04	5.95 E+01	9.17 E+01	8.83 E+01	3.31 E+04	1.37 E+05	9.03 E+00
	Std	2.27E−01	1.27E−04	2.04 E+00	1.92 E+00	2.00 E+00	2.02 E+03	2.41 E+04	1.83 E+00

F7	Ave	1.20E−04	3.74E−05	1.44E−03	2.99E−01	7.95E−03	5.27 E+01	1.91 E+03	1.17E−03
	Std	1.13E−04	3.03E−05	5.54E−04	1.14E−01	2.42E−03	7.61 E+00	7.33 E+02	1.32E−03

F8	Ave	−2.09 E+05	−2.09 E+05	−1.01 E+05	−3.44 E+04	−6.29 E+04	−8.68 E+04	−7.85 E+04	−1.99 E+05
	Std	7.35E−01	1.36 E+00	6.70 E+03	2.48 E+03	9.55 E+03	5.27 E+03	3.02 E+03	1.66 E+04

F9	Ave	0.00 E+00	0.00 E+00	0.00 E+00	5.66 E+03	2.09 E+00	1.92 E+03	3.51 E+03	0.00 E+00
	Std	0.00 E+00	0.00 E+00	0.00 E+00	4.74 E+02	3.15 E+00	1.15 E+02	1.78 E+02	0.00 E+00

F10	Ave	8.88E−16	8.88E−16	8.70E−15	1.10E−07	6.67E−09	1.22 E+01	1.62 E+01	3.97E−15
	Std	0.00 E+00	0.00 E+00	2.17E−15	5.29E−08	1.33E−09	3.43E−01	7.40E−01	2.23E−15

F11	Ave	0.00 E+00	0.00 E+00	0.00 E+00	5.89E−03	1.79E−03	2.87 E+02	1.15 E+03	0.00 E+00
	Std	0.00 E+00	0.00 E+00	0.00 E+00	1.57E−02	6.96E−03	2.17 E+01	2.43 E+02	0.00 E+00

F12	Ave	2.02E−05	2.78E−07	2.65E−01	1.06 E+04	7.01E−01	1.01 E+02	1.34 E+08	1.42E−02
	Std	5.06E−05	3.95E−07	1.85E−02	1.22 E+04	3.05E−02	1.52 E+02	1.59 E+08	3.73E−03

F13	Ave	4.21E−03	4.36E−05	4.86 E+01	1.25 E+03	4.51 E+01	1.01 E+06	5.88 E+08	4.72 E+00
	Std	1.21E−02	7.01E−05	3.40E−01	8.75 E+02	5.29E−01	3.96 E+05	4.43 E+08	1.45 E+00

**Table 6:** Comparison of results on F1–F13 with 1000D.

Function		TSO	HHO	EO	TSA	GWO	SSA	PSO	WOA
F1	Ave	0.00 E+00	5.76E−190	1.08E−72	1.04E−27	3.53E−34	9.88E−03	7.10 E+02	1.15E−168
	Std	0.00 E+00	0.00 E+00	4.12E−72	2.59E−27	5.28E−34	1.10E−02	2.55 E+03	0.00 E+00

F2	Ave	1.96E−231	3.05E−100	2.45E−42	7.52E−18	7.04E−21	1.41 E+01	3.98 E+01	6.29E−103
	Std	0.00 E+00	1.30E−99	2.64E−42	1.09E−17	3.19E−21	4.47 E+00	2.40 E+01	3.45E−102

F3	Ave	0.00 E+00	2.81E−145	2.12E−06	7.99 E+02	1.67E−01	2.21 E+04	8.11 E+04	7.18 E+05
	Std	0.00 E+00	1.54E−144	5.59E−06	1.02 E+03	3.53E−01	1.05 E+04	1.91 E+04	1.23 E+05

F4	Ave	1.49E−229	1.30E−97	5.51E−11	2.86 E+01	1.38E−04	1.96 E+01	3.25 E+01	7.08 E+01
	Std	0.00 E+00	4.59E−97	1.75E−10	9.58 E+00	2.18E−04	2.29 E+00	3.62 E+00	2.99 E+01

F5	Ave	1.15E−01	3.74E−03	9.41 E+01	9.79 E+01	9.70 E+01	7.13 E+02	1.05 E+04	9.73 E+01
	Std	4.32E−01	5.74E−03	3.34E−01	8.33E−01	8.86E−01	4.63 E+02	2.31 E+04	4.64E−01

F6	Ave	1.08E−03	3.41E−05	1.43E−01	1.38 E+01	7.36 E+00	8.23E−03	1.04 E+03	5.30E−01
	Std	2.11E−03	5.76E−05	2.04E−01	9.64E−01	1.16 E+00	8.37E−03	3.06 E+03	1.70E−01

F7	Ave	1.17E−04	4.11E−05	6.99E−04	1.31E−02	1.56E−03	7.61E−01	4.21 E+00	9.37E−04
	Std	1.62E−04	6.24E−05	3.25E−04	4.63E−03	7.36E−04	1.72E−01	7.59 E+00	1.42E−03

F8	Ave	−4.19 E+04	−4.19 E+04	−2.89 E+04	−1.45 E+04	−1.67 E+04	−2.41 E+04	−2.35 E+04	−3.79 E+04
	Std	8.33E−02	3.21E−01	1.54 E+03	7.90 E+02	2.56 E+03	1.51 E+03	1.51 E+03	4.58 E+03

F9	Ave	0.00 E+00	0.00 E+00	0.00 E+00	9.20 E+02	1.49E−01	1.34 E+02	3.14 E+02	0.00 E+00
	Std	0.00 E+00	0.00 E+00	0.00 E+00	1.38 E+02	8.18E−01	3.79 E+01	5.10 E+01	0.00 E+00

F10	Ave	8.88E−16	8.88E−16	7.52E−15	5.96E−12	7.03E−14	5.15 E+00	4.61 E+00	4.44E−15
	Std	0.00 E+00	0.00 E+00	1.23E−15	3.24E−11	4.73E−15	1.25 E+00	3.08 E+00	2.64E−15

F11	Ave	0.00 E+00	0.00 E+00	0.00 E+00	2.71E−03	7.62E−04	1.25E−01	7.31 E+00	0.00 E+00
	Std	0.00 E+00	0.00 E+00	0.00 E+00	6.26E−03	2.94E−03	2.90E−02	2.28 E+01	0.00 E+00

F12	Ave	3.03E−06	3.47E−07	1.89E−03	9.22 E+00	1.93E−01	9.90 E+00	1.02 E+01	6.08E−03
	Std	8.46E−06	5.00E−07	5.68E−03	3.74 E+00	6.19E−02	2.58 E+00	4.20 E+00	3.07E−03

F13	Ave	1.44E−04	1.19E−05	2.12 E+00	1.21 E+01	5.68 E+00	1.54 E+02	4.54 E+02	6.00E−01
	Std	2.32E−04	1.74E−05	1.15 E+00	1.61 E+00	3.89E−01	1.57 E+01	4.27 E+02	3.17E−01

**Table 7:** Comparison of results on F14–F23.

Function		TSO	HHO	EO	TSA	GWO	SSA	PSO	WOA
F14	Ave	9.98E−01	9.98E−01	9.98E−01	8.12 E+00	3.87 E+00	9.98E−01	9.98E−01	1.49 E+00
	Std	2.80E−16	8.98E−11	1.01E−16	4.59 E+00	3.96 E+00	2.08E−16	4.12E−17	1.97 E+00

F15	Ave	3.99E−04	3.54E−04	1.17E−03	1.03E−02	1.71E−03	1.48E−03	1.90E−03	5.35E−04
	Std	2.79E−04	1.65E−04	3.65E−03	2.39E−02	5.08E−03	3.58E−03	5.03E−03	2.82E−04

F16	Ave	−1.03 E+00	−1.03 E+00	−1.03 E+00	−1.03 E+00	−1.03 E+00	−1.03 E+00	−1.03 E+00	−1.03 E+00
	Std	5.61E−16	9.30E−14	6.58E−16	9.65E−03	3.20E−09	8.96E−15	6.78E−16	2.13E−11

F17	Ave	3.98E−01	3.98E−01	3.98E−01	3.98E−01	3.98E−01	3.98E−01	3.98E−01	3.98E−01
	Std	0.00 E+00	1.12E−08	0.00 E+00	6.82E−06	1.64E−07	3.83E−15	0.00 E+00	4.78E−07

F18	Ave	3.00 E+00	3.00 E+00	3.00 E+00	9.30 E+00	3.00 E+00	3.00 E+00	3.00 E+00	3.00 E+00
	Std	1.75E−15	2.51E−09	9.69E−16	2.09 E+01	5.86E−06	3.90E−14	1.39E−15	1.96E−06

F19	Ave	−3.86 E+00	−3.86 E+00	−3.86 E+00	−3.86 E+00	−3.86 E+00	−3.86 E+00	−3.86 E+00	−3.86 E+00
	Std	2.46E−15	6.38E−04	2.70E−15	1.41E−03	2.73E−03	1.84E−14	2.71E−15	2.35E−03

F20	Ave	−3.30 E+00	−3.18 E+00	−3.26 E+00	−3.21 E+00	−3.25 E+00	−3.23 E+00	−3.26 E+00	−3.22 E+00
	Std	4.84E−02	8.68E−02	6.37E−02	1.79E−01	7.38E−02	4.87E−02	6.68E−02	8.34E−02

F21	Ave	−1.02 E+01	−5.22 E+00	−9.81 E+00	−6.78 E+00	−9.65 E+00	−9.06 E+00	−6.98 E+00	−9.81 E+00
	Std	5.68E−15	9.28E−01	1.29 E+00	3.19 E+00	1.54 E+00	2.26 E+00	3.53 E+00	1.29 E+00

F22	Ave	−1.04 E+01	−5.44 E+00	−1.04 E+01	−8.24 E+00	−1.04 E+01	−9.62 E+00	−8.42 E+00	−9.00 E+00
	Std	8.08E−16	1.35 E+00	9.90E−16	3.21 E+00	1.39E−04	2.06 E+00	3.14 E+00	2.54 E+00

F23	Ave	−1.05 E+01	−5.31 E+00	−1.00 E+01	−7.87 E+00	−1.05 E+01	−9.49 E+00	−8.84 E+00	−9.15 E+00
	Std	1.98E−15	9.78E−01	1.65 E+00	3.68 E+00	1.46E−04	2.43 E+00	3.18 E+00	2.79 E+00

**Table 8:** Wall-clock time costs of TSO and other algorithms on 23 benchmarks (unit: s).

Function	TSO	HHO	EO	TSA	GWO	SSA	PSO	WOA
F1	0.0436	0.0792	0.0726	0.0466	0.0549	0.0700	0.0923	0.0365
F2	0.0386	0.0711	0.0698	0.0483	0.0536	0.0679	0.0785	0.0349
F3	0.1878	0.6471	0.2078	0.1938	0.2025	0.2168	0.2187	0.1804
F4	0.0315	0.0765	0.0655	0.0442	0.0486	0.0613	0.0687	0.0286
F5	0.0387	0.1070	0.0723	0.0494	0.0574	0.0679	0.0745	0.0366
F6	0.0313	0.0896	0.0633	0.0434	0.0514	0.0743	0.0732	0.0301
F7	0.0502	0.1198	0.0853	0.0619	0.0690	0.0813	0.0953	0.0468
F8	0.0433	0.1270	0.0738	0.0535	0.0613	0.0789	0.0840	0.0428
F9	0.0341	0.0987	0.0652	0.0474	0.0543	0.0658	0.0785	0.0305
F10	0.0402	0.1096	0.0703	0.0521	0.0558	0.0745	0.0764	0.0354
F11	0.0478	0.1335	0.0753	0.0557	0.0608	0.0805	0.0820	0.0436
F12	0.1055	0.2735	0.1359	0.1154	0.1291	0.1372	0.1494	0.1062
F13	0.1040	0.2881	0.1338	0.1197	0.1350	0.1367	0.1482	0.1064
F14	0.2817	0.7229	0.3301	0.2855	0.2796	0.3102	0.3252	0.2855
F15	0.0337	0.0792	0.0633	0.0281	0.0294	0.0465	0.0651	0.0271
F16	0.0263	0.0619	0.0521	0.0202	0.0211	0.0385	0.0525	0.0195
F17	0.0229	0.0577	0.0501	0.0181	0.0177	0.0343	0.0505	0.0181
F18	0.0220	0.0585	0.0519	0.0175	0.0181	0.0343	0.0520	0.0171
F19	0.0453	0.1182	0.0745	0.0405	0.0420	0.0581	0.0735	0.0397
F20	0.0465	0.1143	0.0739	0.0427	0.0431	0.0604	0.0756	0.0407
F21	0.0739	0.1803	0.1041	0.0680	0.0684	0.0880	0.1028	0.0682
F22	0.0946	0.2264	0.1166	0.0822	0.0844	0.1053	0.1166	0.0820
F23	0.1161	0.2873	0.1502	0.1145	0.1115	0.1313	0.1482	0.1133

**Table 9:** Ranking of results for F1–F7 with varied values of parameter *z* and a.

z	*a*									
	0.1	0.2	0.3	0.4	0.5	0.6	0.7	0.8	0.9	Average
0	61.26	64.72	57.17	55.07	54.46	55.54	58.02	57.00	56.04	57.70
0.01	52.78	49.13	47.09	51.02	46.57	48.15	46.93	48.74	49.30	48.86
0.02	50.13	49.72	54.24	50.89	55.35	45.78	46.65	44.26	45.61	49.18
0.03	55.24	51.96	54.41	48.65	45.33	57.91	51.15	46.11	45.26	50.67
0.04	55.50	52.15	51.50	49.39	47.24	46.37	43.83	46.89	44.74	48.62
0.05	50.74	49.22	47.52	46.11	49.54	43.46	41.83	47.91	45.74	46.90
0.06	53.11	52.50	47.65	46.15	45.96	53.13	48.48	43.63	48.30	48.77
0.07	48.30	50.50	48.50	46.43	51.96	50.91	45.02	46.48	52.70	48.98
0.08	50.57	53.11	43.61	45.02	51.07	45.35	45.17	53.35	46.15	48.15
0.09	51.57	55.91	47.93	50.98	52.35	54.89	52.02	52.50	48.52	51.85
0.1	51.50	51.83	57.41	51.72	54.61	49.33	53.39	43.28	39.83	50.32
Average	52.79	52.79	50.64	49.22	50.40	50.08	48.41	48.20	47.47	

**Table 10:** Ranking of the results for F8–F23 with varied values of parameter *z* and a.

z	*a*									
	0.1	0.2	0.3	0.4	0.5	0.6	0.7	0.8	0.9	Average
0	36.57	43.14	36.14	28.71	38.86	35.86	40.57	33.57	39.00	36.94
0.01	37.00	45.00	34.00	37.00	32.00	30.71	36.00	30.86	34.71	35.25
0.02	41.43	43.29	48.86	44.57	50.29	43.71	40.14	39.14	36.57	43.11
0.03	54.43	39.43	50.86	40.86	34.00	47.86	51.43	44.71	37.71	44.59
0.04	47.57	49.57	49.71	44.71	49.00	42.71	46.14	39.29	35.29	44.89
0.05	52.00	42.14	52.43	58.14	57.57	40.71	43.29	53.29	40.29	48.87
0.06	58.57	51.71	47.00	46.00	49.43	57.43	44.57	39.14	52.00	49.54
0.07	57.86	60.71	57.29	49.14	72.14	62.86	47.86	53.71	48.57	56.68
0.08	63.43	60.86	45.86	56.57	67.43	57.86	62.14	62.43	52.57	58.79
0.09	58.43	73.14	59.57	68.00	69.86	72.29	66.57	60.14	58.43	65.16
0.1	62.71	71.86	73.57	69.14	67.00	65.57	63.14	67.71	54.86	66.17
Average	51.82	52.81	50.48	49.35	53.42	50.69	49.26	47.64	44.55	

**Table 11:** Ranking of results for F1–F23 with varied values of parameter *z* and a.

z	*a*									
	0.1	0.2	0.3	0.4	0.5	0.6	0.7	0.8	0.9	Average
0	72.06	74.16	66.38	66.59	61.28	64.16	65.66	67.25	63.50	66.78
0.01	59.69	50.94	52.81	57.16	52.94	55.78	51.72	56.56	55.69	54.81
0.02	53.94	52.53	56.59	53.66	57.56	46.69	49.50	46.50	49.56	51.84
0.03	55.59	57.44	55.97	52.06	50.28	62.31	51.03	46.72	48.56	53.33
0.04	58.97	53.28	52.28	51.44	46.47	47.97	42.81	50.22	48.88	50.26
0.05	50.19	52.31	45.38	40.84	46.03	44.66	**41.19**	45.56	48.13	46.03
0.06	50.72	52.84	47.94	46.22	44.44	51.25	50.19	45.59	46.69	48.43
0.07	44.13	46.03	44.66	45.25	43.13	45.69	43.78	43.31	54.50	45.61
0.08	44.94	49.72	42.63	39.97	43.91	39.88	37.75	49.38	43.34	43.50
0.09	48.56	48.38	42.84	43.53	44.69	47.28	45.66	49.16	44.19	46.03
0.1	46.59	43.06	50.34	44.09	49.19	42.22	49.13	32.59	33.25	43.39
Average	53.22	52.79	50.71	49.16	49.08	49.81	48.04	48.44	48.75	

**Table 12:** Wilcoxon rank-sum test on F1–F13 with Dim = 30.

TSO vs	HHO		EO		TSA		GWO		SSA		PSO		WOA	
Function	*p*	Win	*p*	Win	*p*	Win	*p*	Win	*p*	Win	*p*	Win	*p*	Win
F1	1.21E−12	+	1.21E−12	+	1.21E−12	+	1.21E−12	+	1.21E−12	+	1.21E−12	+	1.21E−12	+
F2	3.02E−11	+	3.02E−11	+	3.02E−11	+	3.02E−11	+	3.02E−11	+	3.02E−11	+	3.02E−11	+
F3	1.21E−12	+	1.21E−12	+	1.21E−12	+	1.21E−12	+	1.21E−12	+	1.21E−12	+	1.21E−12	+
F4	3.02E−11	+	3.02E−11	+	3.02E−11	+	3.02E−11	+	3.02E−11	+	3.02E−11	+	3.02E−11	+
F5	2.20E−07	+	3.02E−11	+	3.02E−11	+	3.02E−11	+	3.02E−11	+	3.02E−11	+	3.02E−11	+
F6	5.49E−11	+	7.39E−11	−	3.02E−11	+	3.02E−11	+	8.48E−09	+	0.02236	−	3.02E−11	+
F7	1.34E−05	−	1.43E−08	+	3.02E−11	+	6.72E−10	+	3.02E−11	+	3.02E−11	+	1.47E−07	+
F8	4.62E−10	+	3.02E−11	+	3.02E−11	+	3.02E−11	+	3.02E−11	+	3.02E−11	+	3.02E−11	+
F9	**NaN**	=	**NaN**	=	1.21E−12	+	0.160802	+	1.21E−12	+	1.21E−12	+	NaN	=
F10	**NaN**	=	2.71E−14	+	1.09E−12	+	5.65E−13	+	1.21E−12	+	1.21E−12	+	1.08E−09	+
F11	**NaN**	=	**NaN**	=	5.37E−06	+	0.081523	+	1.21E−12	+	1.21E−12	+	0.041926	+
F12	4.20E−10	+	5.97E−09	−	3.02E−11	+	3.02E−11	+	3.02E−11	+	**0.340288**	=	3.02E−11	+
F13	3.02E−11	+	**0.137323**	=	3.02E−11	+	3.02E−11	+	7.22E−06	+	0.030317	+	3.02E−11	+

**Table 13:** Wilcoxon rank-sum test on F1–F13 with Dim = 100.

TSO vs	HHO		EO		TSA		GWO		SSA		PSO		WOA	
Function	*p*	Win	*p*	Win	*p*	Win	*p*	Win	*p*	Win	*p*	Win	*p*	Win
F1	1.21E−12	+	1.21E−12	+	1.21E−12	+	1.21E−12	+	1.21E−12	+	1.21E−12	+	1.21E−12	+
F2	3.02E−11	+	3.02E−11	+	3.02E−11	+	3.02E−11	+	3.02E−11	+	3.02E−11	+	3.02E−11	+
F3	1.21E−12	+	1.21E−12	+	1.21E−12	+	1.21E−12	+	1.21E−12	+	1.21E−12	+	1.21E−12	+
F4	3.02E−11	+	3.02E−11	+	3.02E−11	+	3.02E−11	+	3.02E−11	+	3.02E−11	+	3.02E−11	+
F5	1.37E−03	−	3.02E−11	+	3.02E−11	+	3.02E−11	+	3.02E−11	+	3.02E−11	+	3.02E−11	+
F6	3.59E−05	−	1.75E−05	+	3.02E−11	+	3.02E−11	+	1.70E−08	+	3.02E−11	+	3.02E−11	+
F7	1.52E−03	−	4.62E−10	+	3.02E−11	+	5.49E−11	+	3.02E−11	+	3.02E−11	+	1.86E−06	+
F8	3.16E−05	+	3.02E−11	+	3.02E−11	+	3.02E−11	+	3.02E−11	+	3.02E−11	+	3.02E−11	+
F9	**NaN**	=	NaN	=	1.21E−12	+	7.58E−07	+	1.21E−12	+	1.21E−12	+	**NaN**	=
F10	**NaN**	=	8.64E−14	+	1.00E−12	+	9.19E−13	+	1.21E−12	+	1.21E−12	+	1.22E−08	+
F11	**NaN**	=	NaN	=	6.51E−05	+	**0.160802**	=	1.21E−12	+	1.21E−12	+	**NaN**	=
F12	**1.05E−01**	=	1.41E−09	+	3.02E−11	+	3.02E−11	+	3.02E−11	+	3.02E−11	+	3.02E−11	+
F13	2.96E−05	−	3.02E−11	+	3.02E−11	+	3.02E−11	+	3.02E−11	+	3.02E−11	+	3.02E−11	+

**Table 14:** Wilcoxon rank-sum test on F1–F13 with Dim = 500.

TSO vs	HHO		EO		TSA		GWO		SSA		PSO		WOA	
Function	*p*	Win	*p*	Win	*p*	Win	*p*	Win	*p*	Win	*p*	Win	*p*	Win
F1	1.21E−12	+	1.21E−12	+	1.21E−12	+	1.21E−12	+	1.21E−12	+	1.21E−12	+	1.21E−12	+
F2	3.02E−11	+	3.02E−11	+	3.02E−11	+	3.02E−11	+	3.02E−11	+	3.02E−11	+	3.02E−11	+
F3	1.21E−12	+	1.21E−12	+	1.21E−12	+	1.21E−12	+	1.21E−12	+	1.21E−12	+	1.21E−12	+
F4	3.02E−11	+	3.02E−11	+	3.02E−11	+	3.02E−11	+	3.02E−11	+	3.02E−11	+	3.02E−11	+
F5	1.07E−09	−	3.02E−11	+	3.02E−11	+	3.02E−11	+	3.02E−11	+	3.02E−11	+	3.02E−11	+
F6	4.08E−11	−	3.02E−11	+	3.02E−11	+	3.02E−11	+	3.02E−11	+	3.02E−11	+	3.02E−11	+
F7	1.29E−06	−	3.69E−11	+	3.02E−11	+	3.02E−11	+	3.02E−11	+	3.02E−11	+	7.74E−06	+
F8	**1.67E−01**	=	3.02E−11	+	3.02E−11	+	3.02E−11	+	3.02E−11	+	3.02E−11	+	3.02E−11	+
F9	**NaN**	=	**NaN**	=	1.21E−12	+	1.21E−12	+	1.21E−12	+	1.21E−12	+	**NaN**	=
F10	**NaN**	=	6.12E−14	+	1.21E−12	+	1.21E−12	+	1.21E−12	+	1.21E−12	+	9.16E−09	+
F11	**NaN**	=	**NaN**	=	1.21E−12	+	1.20E−12	+	1.21E−12	+	1.21E−12	+	**NaN**	=
F12	7.04E−07	−	3.02E−11	+	3.02E−11	+	3.02E−11	+	3.02E−11	+	3.02E−11	+	3.02E−11	+
F13	8.88E−06	−	3.02E−11	+	3.02E−11	+	3.02E−11	+	3.02E−11	+	3.02E−11	+	3.02E−11	+

**Table 15:** Wilcoxon rank-sum test on F1–F13 with Dim = 1000.

TSO vs	HHO		EO		TSA		GWO		SSA		PSO		WOA	
Function	*p*	Win	*p*	Win	*p*	Win	*p*	Win	*p*	Win	*p*	Win	*p*	Win
F1	1.21E−12	+	1.21E−12	+	1.21E−12	+	1.21E−12	+	1.21E−12	+	1.21E−12	+	1.21E−12	+
F2	3.02E−11	+	3.02E−11	+	3.02E−11	+	3.02E−11	+	3.02E−11	+	3.02E−11	+	3.02E−11	+
F3	1.21E−12	+	1.21E−12	+	1.21E−12	+	1.21E−12	+	1.21E−12	+	1.21E−12	+	1.21E−12	+
F4	3.02E−11	+	3.02E−11	+	3.02E−11	+	3.02E−11	+	3.02E−11	+	3.02E−11	+	3.02E−11	+
F5	5.87E−04	−	3.02E−11	+	3.02E−11	+	3.02E−11	+	3.02E−11	+	3.02E−11	+	3.02E−11	+
F6	3.50E−09	−	3.02E−11	+	3.02E−11	+	3.02E−11	+	3.02E−11	+	3.02E−11	+	3.02E−11	+
F7	1.86E−03	−	3.02E−11	+	3.02E−11	+	3.02E−11	+	3.02E−11	+	3.02E−11	+	3.37E−05	+
F8	**2.23E−01**	=	3.02E−11	+	3.02E−11	+	3.02E−11	+	3.02E−11	+	3.02E−11	+	3.69E−11	+
F9	**NaN**	=	**NaN**	=	1.21E−12	+	1.21E−12	+	1.21E−12	+	1.21E−12	+	**0.333711**	=
F10	**NaN**	=	2.90E−13	+	1.21E−12	+	1.21E−12	+	1.21E−12	+	1.21E−12	+	7.21E−07	+
F11	**NaN**	=	8.99E−11	+	1.21E−12	+	1.21E−12	+	1.21E−12	+	1.21E−12	+	**0.333711**	=
F12	2.00E−05	−	3.02E−11	+	3.02E−11	+	3.02E−11	+	3.02E−11	+	3.02E−11	+	3.02E−11	+
F13	1.25E−05	−	3.02E−11	+	3.02E−11	+	3.02E−11	+	3.02E−11	+	3.02E−11	+	3.02E−11	+

**Table 16:** Wilcoxon rank-sum test on F14–F23.

TSO vs	HHO		EO		TSA		GWO		SSA		PSO		WOA	
Function	*p*	Win	*p*	Win	*p*	Win	*p*	Win	*p*	Win	*p*	Win	*p*	Win
F14	7.27E−11	+	2.81E−04	−	1.99E−11	+	1.99E−11	+	**5.43E−02**	=	1.80E−05	−	1.99E−11	+
F15	8.30E−08	+	4.84E−08	+	8.43E−09	+	3.94E−08	+	1.10E−08	+	4.27E−08	+	5.06E−08	+
F16	1.07E−09	+	1.49E−04	−	1.41E−11	+	1.41E−11	+	1.39E−11	+	1.43E−06	−	1.40E−11	+
F17	1.21E−12	+	**NaN**	=	1.21E−12	+	1.21E−12	+	5.07E−06	+	**NaN**	=	1.21E−12	+
F18	1.46E−10	+	1.85E−02	−	2.09E−11	+	2.09E−11	+	2.09E−11	+	2.88E−03	−	2.09E−11	+
F19	1.75E−11	+	5.29E−05	+	1.75E−11	+	1.75E−11	+	2.35E−11	+	1.10E−05	−	1.75E−11	+
F20	1.09E−08	+	**3.07E−01**	=	3.07E−08	+	8.39E−08	+	7.12E−10	+	**4.65E−01**	=	1.09E−08	+
F21	1.07E−11	+	**3.81E−01**	=	1.07E−11	+	1.07E−11	+	1.07E−11	+	**1.84E−01**	=	1.07E−11	+
F22	6.43E−12	+	0.019605	−	6.43E−12	+	6.43E−12	+	6.43E−12	+	**2.70E−01**	=	6.43E−12	+
F23	2.14E−11	+	2.20E−02	−	2.14E−11	+	2.14E−11	+	2.14E−11	+	**2.31E−01**	=	2.14E−11	+

**Table 17:** Statistical results of the Wilcoxon rank-sum test.

TSO VS.		F1–F13 (Dim = 30)	F1–F13 (Dim = 100)	F1–F13 (Dim = 500)	F1–F13 (Dim = 1000)	F14–F23	Sum
Wilcoxon's rank-sum test (+/ = /-)	HHO	9/3/1	5/4/4	4/4/5	4/4/5	10/0/0	32/15/15
	EO	8/3/2	11/2/0	11/2/0	12/1/0	2/3/5	42/13/7
	TSA	13/0/0	13/0/0	13/0/0	13/0/0	10/0/0	62/0/0
	GWO	13/0/0	12/1/0	13/0/0	13/0/0	10/0/0	61/1/0
	SSA	13/0/0	13/0/0	13/0/0	13/0/0	9/1/0	61/1/0
	PSO	11/1/1	13/0/0	13/0/0	13/0/0	1/5/4	51/6/5
	WOA	12/1/0	11/2/0	11/2/0	11/2/0	10/0/0	55/7/0

**Table 18:** Statistical results of the Friedman test.

			TSO	HHO	EO	TSA	GWO	SSA	PSO	WOA
F1–F13	Dim = 30	Friedman value	1.54	2.23	3.23	6.54	5.15	6.46	6.15	4.69
		Friedman rank	**1**	2	3	8	5	7	6	4
	Dim = 100	Friedman value	1.65	1.73	3.38	6.31	5.00	6.31	7.54	4.08
		Friedman rank	**1**	2	3	6	5	6	8	4
	Dim = 500	Friedman value	1.65	1.73	4.00	6.54	4.92	6.23	7.54	3.38
		Friedman rank	**1**	2	4	7	5	6	8	3
	Dim = 1000	Friedman value	1.54	1.62	4.08	6.46	5.00	6.23	7.46	3.62
		Friedman rank	**1**	2	4	7	5	6	8	3

F14–F23	Fixed dim	Friedman value	1.75	5.90	2.35	7.30	5.40	4.30	3.60	5.40
		Friedman rank	**1**	7	2	8	5	4	3	5

F1–F23	All dim	Friedman value	1.62	2.48	3.46	6.60	5.08	5.98	6.60	4.18
		Friedman rank	**1**	2	3	7	5	6	7	4

**Table 19:** Comparisons of the best solutions offered by reported optimizers for pressure vessel design.

Algorithm	Optimal values for variables				Optimal cost
	*x*_1_	*x*_2_	*x*_3_	*x*_4_	
DDSCA [50]	0.7782	0.3855	40.3198	176.6389	5888.3366
ISCA [51]	0.8125	0.4375	42.0982	176.6389	6059.7410
MBA [52]	0.7802	0.3856	40.4292	198.4964	5889.3216
CPSO [53]	0.8125	0.4375	42.0912	176.7465	6061.0777
TEO [54]	0.7791	0.3852	40.3698	199.3018	5887.5110
hHHO-SCA [55]	0.9459	0.4471	46.8513	125.468	6393.0927
HPSO [56]	0.8125	0.4375	42.0984	176.6366	6059.7143
MVO [57]	0.8215	0.4375	42.0907	176.7386	6060.8066
AFA [58]	0.8125	0.4375	42.0984	176.6366	6059.7143
**TSO**	**0.7782**	**0.3846**	**40.3196**	**199.9999**	**5885.3327**

**Table 20:** Comparisons of best solutions offered by reported optimizers for tension/compression spring design.

Algorithm	Optimal values for variables			Optimal cost
	*x*_1_	*x*_2_	*x*_3_	
GA3 [59]	0.051989	0.363965	10.890522	0.0126810
CPSO [53]	0.051728	0.357644	11.244543	0.0126747
CDE [60]	0.051609	0.354714	11.410831	0.0126702
DDSCA [50]	0.052669	0.380673	10.0153	0.012688
GSA [24]	0.050276	0.323680	13.525410	0.0127022
hHHO-SCA [55]	0.054693	0.433378	7.891402	0.0128229
AEO [61]	0.051897	0.361751	10.879842	0.0126662
MVO [57]	0.05251	0.3762	10.33513	0.012970
TSO	**0.051642**	**0.355609**	**11.354247**	**0.0126652**

**Table 21:** Comparisons of best solutions offered by reported optimizers for welded beam design problem.

Algorithm	Optimal values for variables				Optimal cost
	*x*_1_	*x*_2_	*x*_3_	*x*_4_	
DDSCA [50]	0.20516	3.4759	9.0797	0.20552	1.7305
HGA [62]	0.205712	3.470391	9.039693	0.205716	1.725236
MGWO-III [63]	0.205667	3.471899	9.036679	0.205733	1.724984
IAPSO [64]	0.205729	3.470886	9.036623	0.205729	1.724852
TEO [54]	0.205681	3.472305	9.035133	0.205796	1.725284
hHHO-SCA [55]	0.190086	3.696496	9.386343	0.204157	1.779032
HPSO [56]	0.20573	3.470489	9.036624	0.20573	1.724852
CPSO [53]	0.202369	3.544214	9.048210	0.205723	1.728024
WCA [26]	0.205728	3.470522	9.036620	0.205729	1.724856
TSO	**0.205729**	**3.470490**	**9.036626**	**0.205729**	**1.724854**

## Data Availability

The data used to support the findings of this study are included within the article.

## References

[1] Wu G. (2016). Across neighborhood search for numerical optimization. *Information Sciences*.

[2] Wu G., Pedrycz W., Suganthan P. N., Mallipeddi R. (2015). A variable reduction strategy for evolutionary algorithms handling equality constraints. *Applied Soft Computing*.

[3] Tang A.-D., Han T., Zhou H., Xie L. (2021). An improved equilibrium optimizer with application in unmanned aerial vehicle path planning. *Sensors*.

[4] Houssein E. H., Emam M. M., Ali A. A. (2021). An efficient multilevel thresholding segmentation method for thermography breast cancer imaging based on improved chimp optimization algorithm. *Expert Systems with Applications*.

[5] Abd Elaziz M., Yousri D., Al-qaness M. A. A., AbdelAty A. M., Radwan A. G., Ewees A. A. (2021). A Grunwald-Letnikov based Manta ray foraging optimizer for global optimization and image segmentation. *Engineering Applications of Artificial Intelligence*.

[6] Xu Y., Huang H., Asghar Heidari A. (2021). MFeature: towards high performance evolutionary tools for feature selection. *Expert Systems with Applications*.

[7] Alweshah M., Khalaileh S. A., Gupta B. B., Almomani A., Hammouri A. I., Al-Betar M. A. (2020). The monarch butterfly optimization algorithm for solving feature selection problems. *Neural Computing & Applications*.

[8] Li Y., Han T., Zhao H., Gao H. (2019). An adaptive whale optimization algorithm using Gaussian distribution strategies and its application in heterogeneous ucavs task allocation. *IEEE Access*.

[9] Wang X., Zhao H., Han T., Zhou H., Li C. (2019). A grey wolf optimizer using Gaussian estimation of distribution and its application in the multi-UAV multi-target urban tracking problem. *Applied Soft Computing*.

[10] Abdel-Basset M., Mohamed R., Chakrabortty R. K., Sallam K., Ryan M. J. (2021). An efficient teaching-learning-based optimization algorithm for parameters identification of photovoltaic models: analysis and validations. *Energy Conversion and Management*.

[11] Hao Q., Zhou Z., Wei Z., Chen G. (2020). Parameters identification of photovoltaic models using a multi-strategy success-history-based adaptive differential evolution. *IEEE Access*.

[12] Lin S.-W., Cheng C.-Y., Pourhejazy P., Ying K.-C., Lee C.-H. (2021). New benchmark algorithm for hybrid flowshop scheduling with identical machines. *Expert Systems with Applications*.

[13] Liu W., Dridi M., Fei H., El Hassani A. H. (2021). Hybrid metaheuristics for solving a home health care routing and scheduling problem with time windows, synchronized visits and lunch breaks. *Expert Systems with Applications*.

[14] Hare W., Nutini J., Tesfamariam S. (2013). A survey of non-gradient optimization methods in structural engineering. *Advances in Engineering Software*.

[15] John Holland H. (1992). *Adaptation in Natural and Artificial Systems*.

[16] Sarker R. A., Elsayed S. M., Ray T. (2014). Differential evolution with dynamic parameters selection for optimization problems. *IEEE Transactions on Evolutionary Computation*.

[17] Koza J. R., Rice J. P. Automatic programming of robots using genetic programming.

[18] Beyer H.-G., Schwefel H.-P. (2002). Evolution strategies – a comprehensive introduction. *Natural Computing*.

[19] Xin Yao X., Yong Liu Y., Guangming Lin G. (1999). Evolutionary programming made faster. *IEEE Transactions on Evolutionary Computation*.

[20] Uymaz S. A., Tezel G., Yel E. (2015). Artificial algae algorithm (AAA) for nonlinear global optimization. *Applied Soft Computing*.

[21] Simon D. (2008). Biogeography-based optimization. *IEEE Transactions on Evolutionary Computation*.

[22] Meng Z., Pan J.-S. (2016). Monkey King Evolution: a new memetic evolutionary algorithm and its application in vehicle fuel consumption optimization. *Knowledge-Based Systems*.

[23] Kirkpatrick S., Gelatt C. D., Vecchi M. P. (1983). Optimization by simulated annealing. *Science*.

[24] Rashedi E., Nezamabadi-pour H., Saryazdi S. (2009). GSA: a gravitational search algorithm. *Information Sciences*.

[25] Wei Z., Huang C., Wang X., Han T., Li Y. (2019). Nuclear reaction optimization: a novel and powerful physics-based algorithm for global optimization. *IEEE Access*.

[26] Eskandar H., Sadollah A., Bahreininejad A., Hamdi M. (2012). Water cycle algorithm - a novel metaheuristic optimization method for solving constrained engineering optimization problems. *Computers & Structures*.

[27] Mirjalili S. (2016). SCA: a Sine Cosine Algorithm for solving optimization problems. *Knowledge-Based Systems*.

[28] Kennedy J., Eberhart R. Particle swarm optimization.

[29] Dorigo M., Di Caro G. Ant colony optimization: a new meta-heuristic.

[30] Mirjalili S., Mirjalili S. M., Lewis Optimizer A. (2014). Grey wolf optimizer. *Advances in Engineering Software*.

[31] Wang G.-G., Deb S., Cui Z. (2019). Monarch butterfly optimization. *Neural Computing & Applications*.

[32] Wang G. G., Deb S., Coelho L. D. S. Elephant herding optimization.

[33] Wang G.-G. (2018). Moth search algorithm: a bio-inspired metaheuristic algorithm for global optimization problems. *Memetic Computing*.

[34] Zhao W., Zhang Z., Wang L. (2020). Manta ray foraging optimization: an effective bio-inspired optimizer for engineering applications. *Engineering Applications of Artificial Intelligence*.

[35] Wang G. G., Deb S., Coelho L. D. S. (2018). Earthworm optimisation algorithm: a bio-inspired metaheuristic algorithm for global optimisation problems. *International Journal of Bio-Inspired Computation*.

[36] Rao R. V., Savsani V. J., Vakharia D. P. (2011). Teaching-learning-based optimization: a novel method for constrained mechanical design optimization problems. *Computer-Aided Design*.

[37] Kumar M., Kulkarni A. J., Satapathy S. C. (2018). Socio evolution & learning optimization algorithm: a socio-inspired optimization methodology. *Future Generation Computer Systems*.

[38] Zhang Y., Jin Z. (2020). Group teaching optimization algorithm: a novel metaheuristic method for solving global optimization problems. *Expert Systems with Applications*.

[39] Askari Q., Saeed M., Younas I. (2020). Heap-based optimizer inspired by corporate rank hierarchy for global optimization. *Expert Systems with Applications*.

[40] Askari Q., Younas I., Saeed Optimizer M. (2020). Political Optimizer: a novel socio-inspired meta-heuristic for global optimization. *Knowledge-Based Systems*.

[41] Alba E., Dorronsoro B. (2005). The exploration/exploitation tradeoff in dynamic cellular genetic algorithms. *IEEE Transactions on Evolutionary Computation*.

[42] Lin L., Gen M. (2009). Auto-tuning strategy for evolutionary algorithms: balancing between exploration and exploitation. *Proceedings of the Soft Computing*.

[43] Wolpert D. H., Macready W. G. (1997). No free lunch theorems for optimization. *IEEE Transactions on Evolutionary Computation*.

[44] Mirjalili S., Lewis A. (2016). The whale optimization algorithm. *Advances in Engineering Software*.

[45] Mirjalili S., Gandomi A. H., Mirjalili S. Z., Saremi S., Faris H., Mirjalili S. M. (2017). Salp Swarm Algorithm: a bio-inspired optimizer for engineering design problems. *Advances in Engineering Software*.

[46] Heidari A. A., Mirjalili S., Faris H., Aljarah I., Mafarja M., Chen H. (2019). Harris hawks optimization: algorithm and applications. *Future Generation Computer Systems*.

[47] Faramarzi A., Heidarinejad M., Stephens B., Mirjalili S. (2020). Equilibrium optimizer: a novel optimization algorithm. *Knowledge-Based Systems*.

[48] Kaur S., Awasthi L. K., Sangal A. L., Dhiman G. (2020). Tunicate Swarm Algorithm: a new bio-inspired based metaheuristic paradigm for global optimization. *Engineering Applications of Artificial Intelligence*.

[49] Tang A., Zhou H., Han T., Xie L. (2021). A modified manta ray foraging optimization for global optimization problems. *IEEE Access*.

[50] Li Y., Zhao Y., Liu J. (2021). Dimension by dimension dynamic sine cosine algorithm for global optimization problems. *Applied Soft Computing*.

[51] Gupta S., Deep K. (2019). Improved sine cosine algorithm with crossover scheme for global optimization. *Knowledge-Based Systems*.

[52] Sadollah A., Bahreininejad A., Eskandar H., Hamdi M. (2013). Mine blast algorithm: a new population based algorithm for solving constrained engineering optimization problems. *Applied Soft Computing*.

[53] He Q., Wang L. (2007). An effective co-evolutionary particle swarm optimization for constrained engineering design problems. *Engineering Applications of Artificial Intelligence*.

[54] Kaveh A., Dadras A. (2017). A novel meta-heuristic optimization algorithm: thermal exchange optimization. *Advances in Engineering Software*.

[55] Kamboj V. K., Nandi A., Bhadoria A., Sehgal S. (2020). An intensify Harris Hawks optimizer for numerical and engineering optimization problems. *Applied Soft Computing*.

[56] He Q., Wang L. (2007). A hybrid particle swarm optimization with a feasibility-based rule for constrained optimization. *Applied Mathematics and Computation*.

[57] Mirjalili S., Mirjalili S. M., Hatamlou A. (2016). Multi-Verse Optimizer: a nature-inspired algorithm for global optimization. *Neural Computing & Applications*.

[58] Baykasoğlu A., Ozsoydan F. B. (2015). Adaptive firefly algorithm with chaos for mechanical design optimization problems. *Applied Soft Computing*.

[59] Coello Coello C. A., Montes E. M. (2002). Constraint-handling in genetic algorithms through the use of dominance-based tournament selection. *Advanced Engineering Informatics*.

[60] Huang F.-z., Wang L., He Q. (2007). An effective co-evolutionary differential evolution for constrained optimization. *Applied Mathematics and Computation*.

[61] Zhao W., Wang L., Zhang Z. (2020). Artificial ecosystem-based optimization: a novel nature-inspired meta-heuristic algorithm. *Neural Computing & Applications*.

[62] Yan X., Liu H., Zhu Z., Wu Q. (2017). Hybrid genetic algorithm for engineering design problems. *Cluster Computing*.

[63] Kumar V., Kumar D. (2017). An astrophysics-inspired Grey wolf algorithm for numerical optimization and its application to engineering design problems. *Advances in Engineering Software*.

[64] Ben Guedria N. (2016). Improved accelerated PSO algorithm for mechanical engineering optimization problems. *Applied Soft Computing*.

